# The relationship between food habits and physical activity and the IQ of primary school children

**DOI:** 10.1186/s41043-024-00522-6

**Published:** 2024-02-20

**Authors:** Alireza Khadem, Maryam Nadery, Sahar Noori, Rasool Ghaffarian-Ensaf, Abolghassem Djazayery, Ariyo Movahedi

**Affiliations:** 1 Department of Nutrition, Science and Research Branch, Islamic Azad University, Tehran, Iran; 2 Department of Dietetics and Nutrition, Robert Stempel College of Public Health and Social Work, Florida International University, Miami, Florida, USA; 3 Department of Community Nutrition, School of Nutritional Sciences and Dietetics, Tehran University of Medical Sciences (TUMS), Tehran, Iran

**Keywords:** IQ, Food habits, Physical activity, Primary school children

## Abstract

**Background:**

Children's intelligence quotient (IQ) is influenced by various environmental and genetic variables. The current study aimed to determine how children's dietary choices and physical activity levels correlated with their IQ.

**Methods:**

A total of 190 students (111 girls and 79 boys) between the ages of 8 and 10 were chosen randomly for this cross-sectional research. For all children, questionnaires were utilized to gather information on their anthropometry, socio-economic position, food habits, and 24-h memory. Children's physical activity questionnaire (CPAQ) was also used to gauge their level of physical activity. Raven's color progressive intelligence test was also used to gauge children's IQ. All the questions may be filled out online with the assistance of parents. SPSS software was used to gather and evaluate the generated data.

**Results:**

Of 190 respondents, 79 (41.6%) are males, and 111 (58.4%) are girls. The results of the study showed that, a positive correlation between children's IQ and physical activity (*P* = 0.017, *r* = 0.17), if this relationship was not seen by gender. In addition, a positive correlation was observed between the IQ and food habits scores in all children (*P* = 0.001, *r* = 0.24), as well as by gender, that is, male (*P* = 0.04, *r* = 0.23) and female (*P* = 0.006, *r* = 0.26), which indicates that children with better food habits were associated with higher IQ.

**Conclusion:**

It was shown that elementary school children's IQ, food habits, and degree of physical activity are all positively correlated.

## Introduction

Intelligence is a fundamental talent whose change or lack has the greatest impact on human life [1]. Intelligence is a mental ability and includes various abilities such as reasoning, planning, problem-solving, abstract thinking, language use, and learning [2]. IQ is a score for the relative evaluation of intelligence and a standard for a person's current performance ability and does not necessarily determine the future [3]. Intelligence is a multifactorial trait that is influenced by countless genetic and environmental factors [3]. Most studies estimate that the heritability of IQ is between 30 and 70% [4]. The strongest evidence about the role of heredity in IQ was shown in comparison with twins; the IQ scores of identical twins were more similar to each other than the IQ scores of non-identical twins [5, 6]. However, intelligence is influenced by environmental factors, including nutrition and physical activity [7].

Nutrition plays an important role in brain development and function. Nutrients provide components that play a key role in cell proliferation, neurotransmitter, and hormone metabolism and are important components of the enzyme system in the brain [8]. Nutrition is one of the factors affecting the cognitive development of children [8]. On the one hand, school age is one of the sensitive ages when sustainable food habits are formed; on the other, primary school students are one of the most vulnerable groups. Their proper physical growth and health services are the foundation for increasing their learning capacity and success in the future [9]. According to research that looked at the connection between 8-year-olds' IQ and their eating patterns and behaviors [10], they discovered that a good diet may be linked to a slight increase in IQ in late childhood [10]. Also, according to research conducted in 23 regions across the nation, children's health is seriously compromised by an idle lifestyle and poor diet [11]. Similarly, young children in Iran have been shown to consume more junk food each week than they do primary meals [12]. Children and teens have different food habits from people of other ages, and those in this age range frequently skip meals, consume snacks and prepared foods, and adhere to unhealthy diets [13]. People acquire their primary food habits and lifestyles at this age, and children's dietary practices, physical activity level, and way of life impact their physical and mental growth [14].

The advantages of physical activity for brain health and function have been demonstrated throughout the past few decades [15]. Doing sports and motor activities is one of the finest methods to expand the brain's capacities and create an ideal environment for optimal learning [16]. Regular participation in sports has also been linked to an increase in people's cognitive function [17]. Play and physical exercise are considered critical factors in children's executive function and natural development [18]. Physical exercise among children is crucial for growth and development, enhancing health, and preventing illnesses [19, 20]. Different mechanisms have been proposed to investigate the impact of physical activity on cognitive performance and IQ [21, 22]. Including these physiological mechanisms: increasing cerebral blood flow in the brain, which shows that moderate- and high-intensity physical exercises significantly increase blood flow in the brain, and increases the supply of necessary nutrients; changes in the release of neurotransmitters including increased levels of norepinephrine and its precursors, epinephrine (adrenaline A) and serotonin after exercise; as well as structural changes in the central nervous system that occur as a result of physical activity in the body [23–25].

Knowing unhealthy eating behaviors will help us identify ways to change or replace them in society with the best nutritional practices. Only a few small-scale studies have been done in this area worldwide, and most of them only looked at one of these characteristics concerning children's IQ levels, producing conflicting results. Based on the studies done, the current study is the first of its sort in the nation. The materials that have been supplied indicate that the purpose of this study is to determine how habits and physical exercise affect primary school students' IQ so that the appropriate preparation may be made to enhance the condition of the kids if there is a link between the variables.

## Methods and materials

### Study population

The participants in this cross-sectional study aged 8–10 years primary school children of Dorud city, who were selected, and questionnaires were provided online after the necessary coordination with education and training. Before filling the questionnaires, the consent form was given to the children's parents, and they all signed it. In the current study which was performed in 2021–2020, students from the second (2722 individuals, 1267 females, and 1455 boys) and third (2459 people, 1190 girls, and 1269 boys) grades in Dorud city of Iran make up the statistical population of this research. The Cochran–Morgan formula, determined by considering the 90% quantile as stated below, was utilized for computation because the statistical population's size was known with confidence. The number *N* represents the size of the community; the combined number of male and female pupils in the locations mentioned earlier who are of the target age is 5181. The sample volume will be determined with the 164 samples and the calculation above.

$$n = \frac{{\frac{{z^{2} pq}}{{d^{2} }}}}{{1 + \frac{1}{N}\left( {\frac{{z^{2} pq}}{{d^{2} }} - 1} \right)}} \to n = \frac{{\frac{{1.3^{2} 0.5 \times 0.5}}{{0.05^{2} }}}}{{1 + \frac{1}{5181}\left( {\frac{{1.3^{2} 0.5 \times 0.5}}{{0.05^{2} }} - 1} \right)}}n = 164$$

These individuals were chosen randomly from among the students who met the requirements to participate in the current research, and the calculation methods with the highest results were chosen. A total of 190 participants were added to the research after sampling. All procedures involving human subjects were approved by the ethical Iran National Committee for Ethics in Biomedical Research with the following identification: IR.IAU.SRB.REC.1400.118.

### Assessment of socio-demographic characteristics and anthropometric

A standard online socio-demographic questionnaire was used to collect information on age, gender, parents' educational and occupational status, and economic status. Also, anthropometric information, including height and weight, was asked through this questionnaire. Anthropometrical z-scores information of children, including the weight-for-age (WAZ), height-for-age (HAZ), and body mass index (BMI)-for-age (BAZ), were added using Anthro Plus V.1.04 software of the World Health Organization (WHO). All of the data were categorized based on the WHO children growth standards guideline.

### Assessment of food habits and physical activity

The dependable validity questionnaire gathers data on food habits (IAU-FHQ81). The Likert scale format, which has four options—always, frequently, sometimes, and never (Scores 4–1)—was used to score the 81 items in this survey. In this questionnaire, a number of questions were given a reverse score, and finally, after summing up the entire questionnaire, they were classified into four categories of poor, average, good, and very good eating habits based on the total score: < 139 for poor, 140–151 for moderate, 152–163 for good, and < 164 for very good. Jalilian Fard et al. evaluated this questionnaire's validity and reliability. The questionnaire's questions were internally consistent, as evidenced by Cronbach's alpha of 0.78 [26].

Children's physical activity questionnaire (CPAQ) was designed by Kowalski et al. This questionnaire contains nine questions that the participants answered on a 5-point Likert scale. Considering that the scoring of this questionnaire is based on the average, the range of scores is between 1 and 5, and the closer the average is to 5, it indicates the high level of physical activity of children. The average score on this questionnaire is 3. Wilk et al. also examined it and used factor analysis, which showed that all factor loadings were over 5%. The construct's validity and reliability were established (0.86 for Cronbach's alpha and above 5%, respectively) [27]. When Dehnavi and Tharvati examined the validity and reliability of this survey in the nation, their findings indicated that its validity was accepted with a likelihood of acceptance and that its internal reliability was 0.67 [28]. Sabzevari et al. conducted research throughout the first phase. Its reliability and validity were validated with a Cronbach's alpha of 0.78 [29].

### Assessment of IQ level status

In this study, to evaluate the level of intelligence, Raven's IQ test for children or the Raven's color progressive matrices (CPM) was used, which is one of the tools for cognitive evaluation or measuring children's visual intelligence. Raven's non-verbal intelligence test has been used all over the world and has been cited as an effective tool for decades. In this test, the child's potential ability to create logical connections between stimuli is used. Raven's intelligence test for children consists of three sets of items A, AB, and B, each of which has 12 items. This test has a total of 36 pictures and is applicable for children aged 5–11 years. In Raven's IQ test, the subject must find the cut part of the original or sample image from among the lower images. Therefore, the subject first looks carefully at the top image and then chooses the desired image from among the six bottom images. The matrices of this test gradually become more difficult [30, 31]. Each correct answer is given 1 point. Taking into account the total scores and age of the subject, his percentage rank is determined based on the standard table. Then, using the percentage score, the child's IQ can be determined.

### Statistical analysis

Kolmogorov–Smirnov test was used to check the normality of the variables. Mean (standard deviation) was used to describe quantitative variables according to conditions, and frequency (percentage) was used for qualitative variables. Independent *t*-test was used to compare the mean of quantitative results between the two studied groups, and Chi-square test was also used to compare the qualitative factors between the two groups. Pearson's correlation was used to determine the relationship between IQ with physical activity and food habits score based on gender. Statistical analyses were done using SPSS version 26.0 (SPSS, Chicago, IL, USA). *P* values less than 0.05 were considered statistically significant.

## Results

### Study population characteristics

The findings revealed that among the 190 participants frequency (%), 79 (41.6%) were men, and 111 (58.4%) were women. Regarding educational level, 87 (45.8%) respondents are in the third grade, and 103 (54.2%) are in the second grade. Parental education is most frequently related to diploma and sub-diploma levels (father 141 (74.2%) and mother 148 (77.9%)). In addition, the highest frequency in terms of mother's occupation is related to housewives, 173 (91.1%) people, while the highest frequency in terms of father's occupation is related to workers, 106 (55.8%) people. Among the participants, 79 (41.6%) had a moderate economic status.

### General characteristics of the study population

The baseline characteristics of the study participants are presented in Table 1. As shown in this table, the mean and standard deviation (SD) age, weight, height, BMI-for-age (BAZ), height-for-age (HAZ), and weight-for-age (WAZ) of participants were 9.23 (0.68) years, 28.43 (6.7) kg, 129.15 (8.4) cm, 0.10 (1.4), − 0.74 (1.4), and − 0.33 (1.3), respectively. Moreover, the mean (SD) IQ, physical activity, and food habits score of participants were 123.29 (12.9), 3.08 (0.73), and 151.2 (11.92), respectively.

**Table 1:** Baseline characteristics of study participants according to mean and standard deviation

Variables	Min	Max	Mean ± SD
Age (years)	8	10	9.23 ± 0.68
Weight (kg)	18	59	28.43 ± 6.7
Height (cm)	90	150	129.15 ± 8.4
WAZ	− 3.4	3.81	− 0.33 ± 1.3
HAZ	− 7.5	4.03	− 0.74 ± 1.4
BAZ	− 4.1	5.8	0.10 ± 1.4
IQ score	70	144	123.29 ± 12.9
Physical activity level	1.56	4.78	3.08 ± 0.73
Food habits score	123	195	151.2 ± 11.92

Quantitative variables were reported as mean ± SD

BAZ, BMI-for-age; CM, centimeter; HAZ, height-for-age; IQ, intelligence quotient; kg, kilogram; and WAZ, weight-for-age

**Fig. 1:**
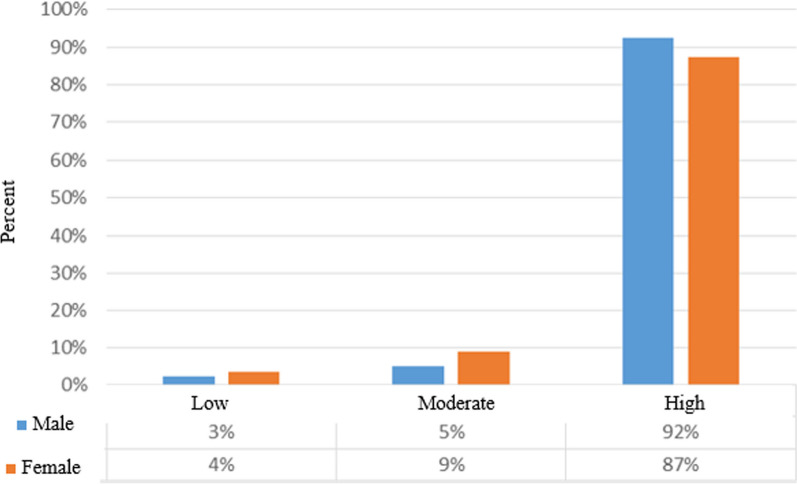
Comparison of the frequency of IQ score of individuals based on gender

After calculating the IQ described in the methods, people were classified into three groups including children with high IQ (110 <), moderate (90 ≤ IQ ≤ 110), and low (< 90). The results obtained from the present study showed that most of the participants had high IQ, and there is an equal frequency distribution between the two groups of male 92% and female 87% in terms of IQ scores (Fig. 1)**.**

**Fig. 2:**
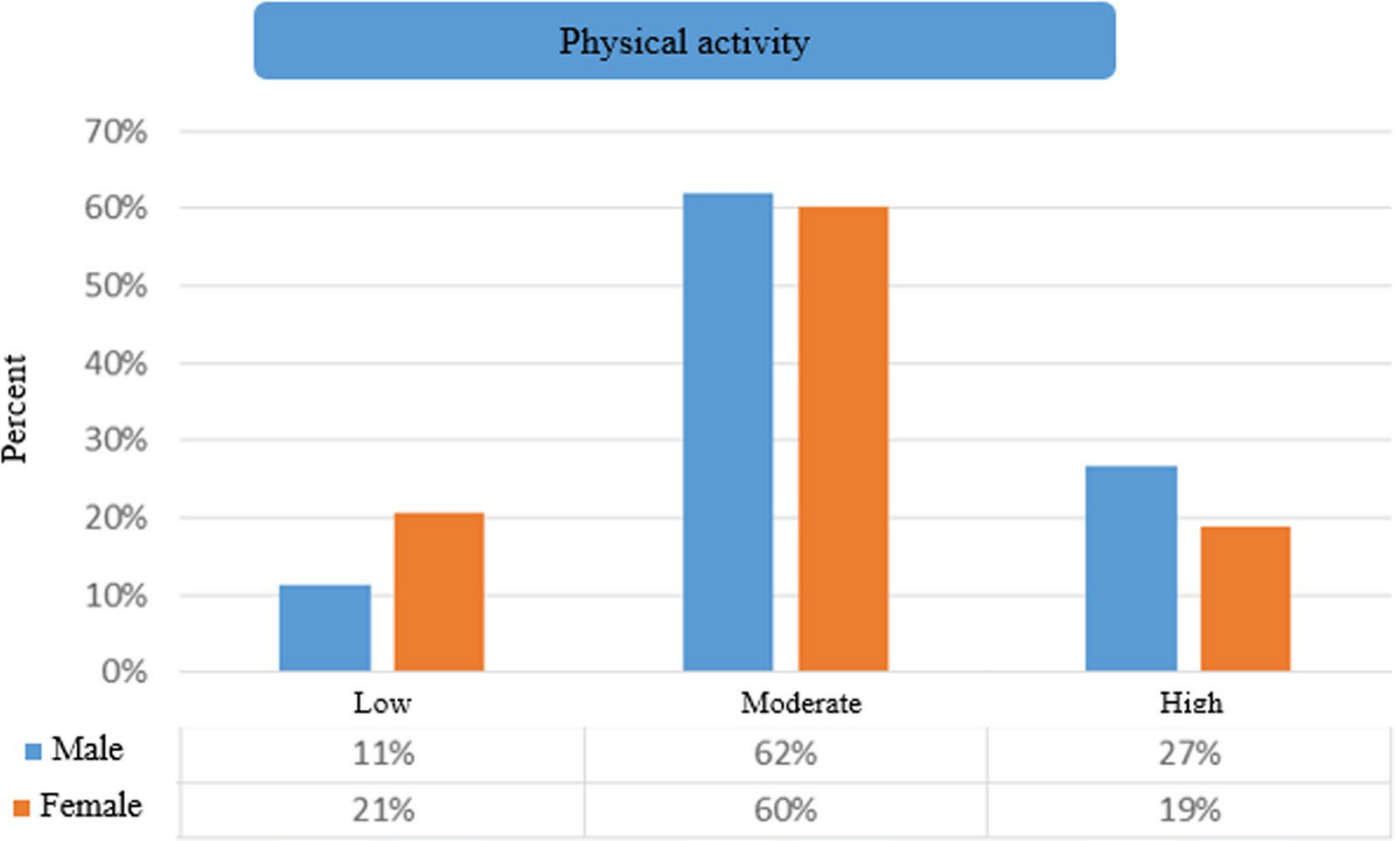
Comparison of the frequency of physical activity levels of individuals based on gender

In terms of physical activity level, most of the people are at a moderate level, so that the male 62% and female 60% of them (Fig. 2).**.**

**Fig. 3:**
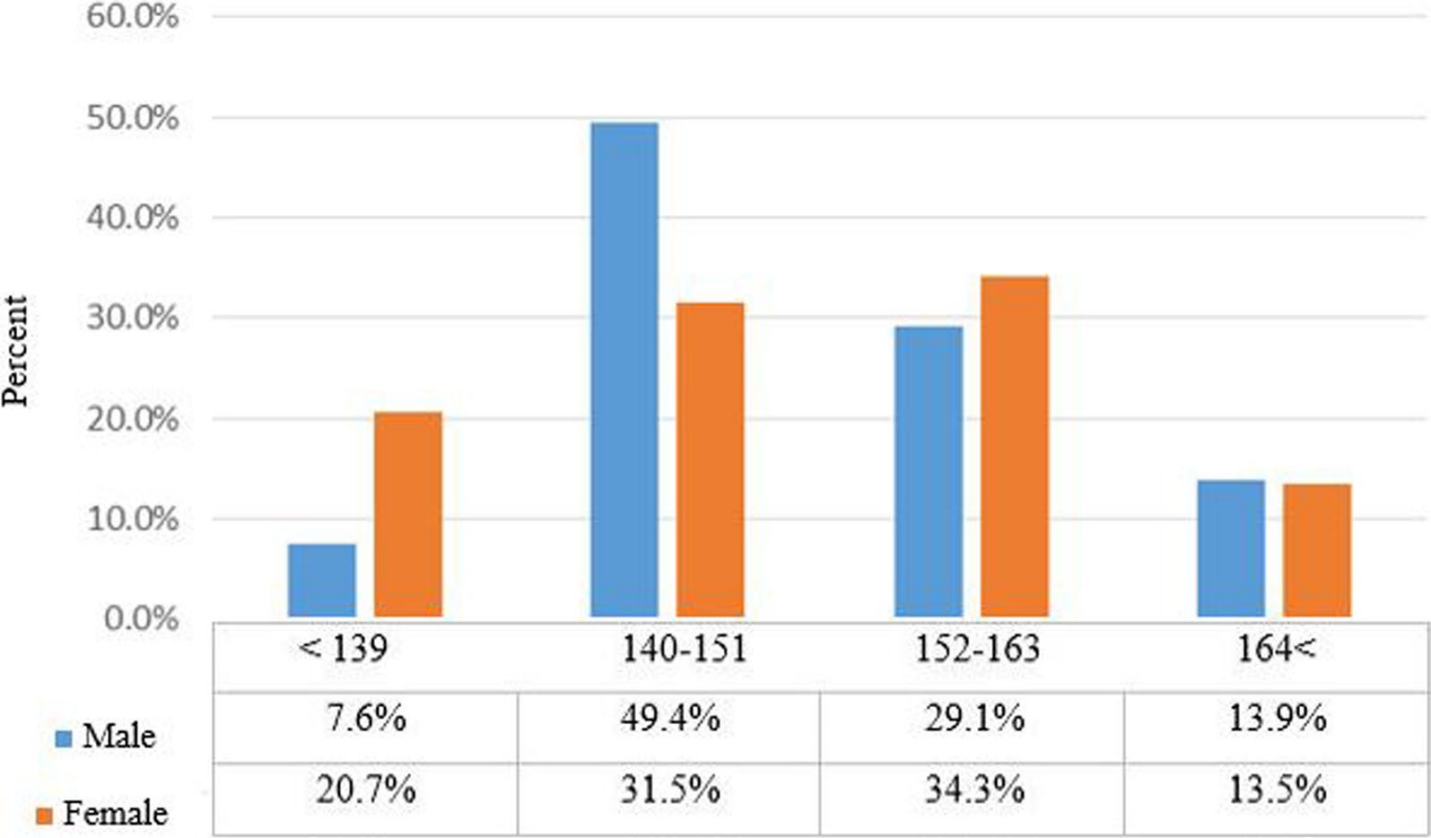
Comparison of the frequency of food habits scores of individuals based on gender

According to Pearson's Chi-square test, we find that there is no significant difference between males and females in terms of the level of physical activity, and the distribution of frequency in the level of physical activity is the same in gender (*P* = 0.16) (Fig. 2). As shown in Fig. 3, according to the cut points of the food habits score (<139, 140-151, 152-163, 164<), which respectively indicate the state of poor, moderate, good, and very good food habits, it was shown that the score of food habits of most people was 140–151 (moderate), and there is no significant difference in terms of frequency distribution between the two groups of male 49.4% and female 31.5%.

### The IQ score, physical activity, and food habits score among the participants based on gender

According to Table 2, a significant mean difference was observed between the two groups of males and females in terms of physical activity, so that it was more in males than females (*P* = 0.041). If no significant mean difference was observed in terms of IQ and food habits scores of both male and female groups (*P* > 0.05).

**Table 2:** Comparing the IQ score, physical activity level, and food habits score among the participants based on gender

Variables	Gender	*N*	Mean ± SD	*P* value
IQ score	Male	79	13.2 ± 124.6	0.22
	Female	111	12.7 ± 122.1	
Physical activity level	Male	79	0.7 ± 3.2	**0.04**
	Female	111	7/0 ± 3	
Food habits score	Male	79	11.2 ± 151.4	0.85
	Female	111	12.4 ± 151.1	

Bold values are significant

Quantitative variables were reported as mean ± SD

*P* values resulted from the analysis of independent t-test for continuous variables

*P* value < 0.05 was considered significant

### The association between the IQ and physical activity, food habits score

In Table 3, Pearson's correlation test showed that there is a weak positive correlation between children's IQ and physical activity level (*p* = 0.017, *r* = 0.17), if this relationship was not seen by gender. In addition, a positive correlation was observed between the IQ and food habits scores in all children (*p* = 0.001, *r* = 0.24), as well as by gender, that is, male (*p* = 0.04, *r* = 0.23) and female (*p* = 0.006, *r* = 0.26), which indicates that children with better food habits were associated with higher IQ.

**Table 3:** Correlation between IQ and physical activity level, food habits score

IQ score		Total	Male	Female
Physical activity level	*r*	0.17	0.17	0.14
	*p* value	**0.017**	0.1	0.1
Food habits score	*r*	0.24	0.23	0.26
	*p* value	**0.001**	**0.04**	**0.006**

Bold values are significant

*r*: correlation coefficient

*p* value < 0.05 was considered significant

### The association between the IQ and WAZ, HAZ, and BAZ

In Table 4, Pearson's correlation test showed that there is a positive correlation between IQ and HAZ (*p* = 0.04, *r* = 0.15) in all children. This relationship based on gender was seen only in the female group HAZ (*p* = 0.01, *r* = 0.23). In other variables, there was no significant relationship with IQ, either in the whole children or by gender.

**Table 4:** Correlation between IQ and WAZ, HAZ, and BAZ

IQ score		Total	Male	Female
WAZ	*r*	0.22	0.18	0.22
	*p* value	0.16	0.12	0.15
HAZ	*r*	0.15	0.04	0.23
	*p* value	**0.04**	0.61	**0.01**
BAZ	*r*	0.17	0.20	0.12
	*p* value	0.12	0.07	0.11

Bold values are significant

BAZ, BMI-for-age; HAZ, height-for-age; IQ, intelligence quotient; *r*, correlation coefficient; and WAZ, weight-for-age

*p* value < 0.05 was considered significant

## Discussion

In the present study, we investigated the association between dietary habits and physical activity with IQ of primary school children. The findings indicated that food habits correlate with IQ scores for both genders of primary school students. Boys and girls did not differ in IQ or overall eating preferences. However, there was no difference between boys and girls; most kids had high IQs. According to the study's findings, children's IQ and nutritional habits are positively and significantly correlated in both sexes, meaning that kids with better food habits have high IQs. This case's finding is consistent with some other research that found a connection between children's IQ and their eating and physical activity patterns that are beneficial [32, 33]. According to the study by Abiri et al., total calorie consumption and physical activity did not substantially affect IQ [34]. The difference in sample size between the two studies might cause this variation. Additionally, Northstone et al. (2011) concluded that there is evidence linking poor nutrition and food habits in early childhood linked with excessive fat, sugar, and processed food to a slight decline in IQ in late childhood [10]. However, a slight rise in IQ may be related to healthy eating practices and high nutrient intake. According to Ghazi et al. (2012), children who do not eat breakfast are 7.4 times more likely to have a poor IQ than children who do [33]. Eating breakfast is, therefore, seen to be a healthy food habit.

The findings indicated that boys usually engage in more physical activity than girls, which is consistent with other research [35, 36]. There are several possible explanations for this result, why girls are less active than boys in terms of physical activity. As studies have shown that girls participate less in organized sports [37], may also perceive less enjoyment when participating in physical education [38]. Another explanation related to this difference can be due to biological reasons, which shows that physical activity levels between boys and girls decrease after adjusting for sexual maturity, and lower levels in girls may be related to puberty at an earlier chronological age [39].

In the current study, it was also demonstrated that children's overall levels of physical activity had a direct correlation with their IQ, and our findings were consistent with those of studies by Kholy (2015) and Makharia (2016) [32, 40]. This may be explained by the fact that exercise promotes the growth of nerve cells by increasing neurogenesis, oxygenation, and levels of neurotransmitters like dopamine. Intense exercise also lowers stress and enhances mental performance [41]. Boys engaged in greater physical activity than girls, but there was no correlation between gender and IQ in terms of physical activity. Our findings were consistent with Abiri's study, which revealed no discernible connection between girls' IQ and physical activity levels [34]. Exploratory activities give kids the power to develop vital motor skills between the ages of 7 and 10. As a result, it has been demonstrated how physical exercise affects children's mental capacity and intellect. However, our study further demonstrated that children's gender did not affect how much physical exercise was associated with their IQ. According to research, engaging in physical activity increases general health and performance and does not negatively impact academic achievement [34]. The diverse methodologies employed to measure physical activity in this study may, in part, account for the lack of a link between IQ and physical activity by gender. The participants' varied ages may have also impacted the comparability of the research.

The findings of the present study show a positive and significant correlation between HAZ and IQ in all children and also by gender only in girls, which is in line with the results of Kanazawa et al. (2009) and Beauchamp et al. (2011), so that with increasing height, children's IQ has been higher [42, 43]. But it is against the results of Feyzpour et al. (2019) [44]. Female children have reached physical growth faster, and on the other hand, in childhood, they have higher practical, verbal, and cognitive intelligence compared to boys, but these differences disappear with age [45, 46]. The findings of this research were also in line with the results of Feyzpour et al. (2019) and Harris et al. (2016) that there was no significant relationship between WAZ and BAZ with IQ [44, 47]. On the other hand, Sohrabi et al. (2015) reported that low IQ score with weight and a higher BMI has been associated [48]. The difference in the findings of this study with other researches can be caused by the difference in socio-economic status and physical activity that affects the relationship between obesity and intelligence [49].

The present study has several limitations. This study is cross-sectional and prevents inference of causality. In addition, due to the fact that the questionnaires were provided to the audience online, there were problems in answering some tests by children and parents, as well as in the cooperation of schools. In addition, the questionnaires were self-reported, which depended on memory, and, on the other hand, were prone to bias. The present study did not determine the heredity of intelligence since the Raven's examination was not administered to parents or grandparents. Despite the mentioned limitations, our study has strong points, including the number of participating and examining the study in two gender groups, as well as the use of validated questionnaires for IQ, food habits, and physical activity.

## Conclusion

In summary, this study shows that there is a positive and significant correlation between children's IQ and food habits in both genders. It also shows that males were more physically active than females, and the level of physical activity of all children is directly related to their IQ, but there is no correlation between gender and IQ in terms of physical activity. However, further research is necessary to confirm these concepts in this field.

## Data Availability

The data that confirm the findings of this study are available from Ariyo Movahedi, but restrictions apply to the availability of these data, which were used under license for the current study, so they are not publicly available. Data are available from the authors upon reasonable request and with permission from Ariyo Movahedi.
